# Description of a combination of Buck's technique and discectomy for spondylolysis with superior-level disc herniation: A case report

**DOI:** 10.1016/j.ijscr.2025.111159

**Published:** 2025-03-14

**Authors:** Shahin Naghizadeh, Maryam Zohrabi-Fard, Saeed Oraee-Yazdani

**Affiliations:** a Functional Neurosurgery Research Center, Research Institute of Functional Neurosurgery, Shohada Tajrish Neurosurgical Center of Excellence, Shahid Beheshti University of Medical Sciences, Tehran, Iran; b Student Research Committee, School of Medicine, Shahid Beheshti University of Medical Sciences, Tehran, Iran

**Keywords:** Spondylolysis, Disc herniation, Buck's technique, Discectomy, Case report

## Abstract

**Introduction and importance:**

Spondylolysis is a pars interarticularis defect often associated with instability and pain. While commonly involving the inferior level, it can rarely present with disc degeneration or herniation at the superior level. Such coexistence poses unique biomechanical and clinical challenges, particularly in younger patients who require solutions that preserve spinal motion and minimize future degeneration. This report highlights a novel combined surgical approach while preserving spinal motion, addressing both pathologies.

**Case presentation:**

A 27-year-old male presented with chronic low back pain and newly exacerbated radiculopathy. Imaging revealed bilateral L5 spondylolysis and a concomitant right-sided L4/L5 disc herniation compressing the L5 nerve root without significant spondylolisthesis. Conservative management was unsuccessful. A combined surgical approach using Buck's technique for the pars defect and discectomy at L4/L5 was performed. This strategy stabilized the spine, alleviated nerve compression, and preserved spinal motion. Postoperative imaging confirmed defect resolution and restored alignment without neurological deficits. At one-year follow-up, the patient reported marked pain relief and a return to normal activities.

**Clinical discussion:**

This rare coexistence required a tailored surgical strategy balancing motion preservation and structural stability. Buck's technique effectively stabilized the pars defect, while discectomy alleviated nerve root compression. This approach avoided fusion-related risks, ensuring long-term spinal functionality and reducing the likelihood of adjacent segment degeneration.

**Conclusion:**

The rare coexistence of L5 spondylolysis with L4/L5 disc herniation presents unique challenges. This case demonstrates that the first reported combination of Buck's technique and discectomy effectively resolves spinal instability and nerve compression while preserving motion.

## Highlights

• First reported use of Buck's technique with discectomy for L5 spondylolysis
• Tailored, motion-preserving approach for managing rare spinal pathologies
• The one-year follow-up shows successful recovery and resolution of dual spinal issues.

## 1 Introduction

Spondylolysis, a bony defect of the pars interarticularis, results from congenital or acquired stress fractures due to chronic trauma [1]. It commonly affects the lower lumbar spine, particularly in adult athletes involved in hyperextension and rotation sports, such as gymnastics and tennis. Although typically asymptomatic, when symptomatic, it can cause significant pain and is a frequent cause of low back pain in adolescents and athletes [1]. If left untreated, spondylolysis can significantly impact the quality of life, as more than 50 % of cases progress to spondylolisthesis [1,2]. This progression can lead to debilitating symptoms such as radiating pain, numbness, or weakness in the lower extremities [1]. The condition also decreases the lumbar spine's segmental stability, increasing the load on the affected disc at adjacent levels, thereby accelerating adjacent segment degeneration (ASD) [3]. For young athletes, these physical limitations, combined with chronic pain and restricted activity, can have profound effects on mental health, potentially derailing overall well-being [4]. The treatment of spondylolysis ranged from conservative methods to surgical options; however, complications such as restricted range of motion, ASD, and postoperative instability can significantly impact long-term spinal function and quality of life.

Here, we present a unique case of a 27-year-old patient diagnosed with L5 spondylolysis accompanied by superior-level disc herniation, which poses distinct clinical challenges. To our knowledge, this is the first reported instance of utilizing Buck's technique in combination with discectomy for this condition. This innovative approach provides a promising solution for addressing concurrent spinal instability and disc pathology, setting a new precedent in the surgical management of such complex cases.

## 2 Presentation of case

This case report adheres to the Surgical CAse REport (SCARE) 2023 guidelines [5]. In the present study, a 27-year-old male presented with a five-year history of chronic low back pain, notably aggravated by activity and positional changes, recently exacerbated by the onset of radicular pain unresponsive to medical treatment. The sharp pain radiated to both forefeet through the posterior aspect of the thighs, with a Visual Analog Scale (VAS) score of 8. A comprehensive evaluation of the patient's history revealed no significant trauma, falls, or involvement in martial arts that could have contributed to the condition. On physical examination, the patient demonstrated normal motor strength but exhibited a decreased Achilles reflex. The patient had no prior neurological symptoms, such as sensory deficits, significant weakness, or gait abnormalities.

**Fig. 1:**
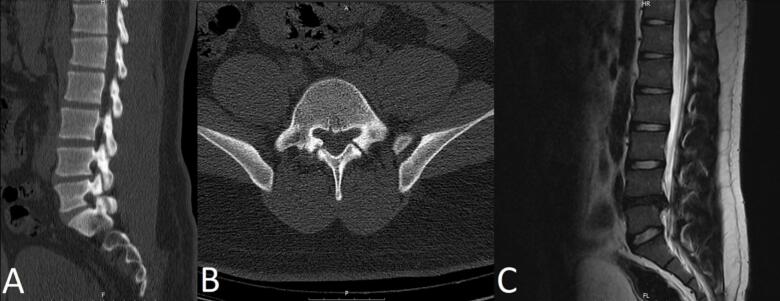
Preoperative imaging: Imaging of the lumbar spine showing bilateral spondylolysis at L5 (A, B) on preoperative CT scan and right-sided L4/L5 disc protrusion compressing the L5 nerve root (C) on MRI. No evidence of spondylolisthesis or endplate signal changes was observed.

Imaging studies revealed bilateral spondylolysis at the L5 level on Computed Tomography (CT) scans, characterized by defects in the pars interarticularis on both sides, visible as discontinuities on sagittal views (Fig. 1A) and gaps on axial views (Fig. 1B), with no evidence of significant spondylolisthesis as vertebral alignment was preserved. Magnetic Resonance Imaging (MRI) findings (Fig. 1C) demonstrated a moderate reduction in L4/L5 disc height, indicative of degenerative changes, and a right-sided disc protrusion compressing the L5 nerve root within the neural foramen. Notably, vertebral endplates showed no signal changes such as Modic changes, suggesting no acute inflammatory or significant degenerative processes in the vertebral bodies. Additionally, vertebral alignment remained intact on MRI, further confirming the absence of spondylolisthesis. These imaging findings highlight the co-occurrence of L5 spondylolysis and L4/L5 disc herniation, explaining the patient's chronic low back pain and radiculopathy.

The patient underwent a combined surgical approach using Buck's technique and discectomy to address L5 spondylolysis and L4/L5 disc herniation. Following a standard posterior midline exposure, a laminectomy and discectomy were performed at the L4/L5 level to remove herniated disc material compressing the nerve root, achieving adequate decompression. The bilateral pars defects at the L5 level were then skeletonized, and fibrous tissue and sclerotic bone within the defects were removed with a curette until a bleeding bony surface was prepared.

**Fig. 2:**
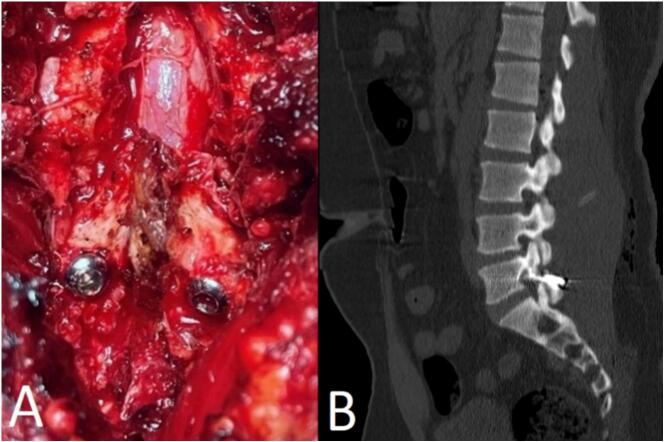
Intraoperative and postoperative imaging. Intraoperative photograph showing the successful placement of bilateral cortical screws across the pars defects at the L5 level using Buck's technique, with autologous bone graft packed around the screws (A). Postoperative sagittal CT scan demonstrating proper screw placement across the L5 pars defects with intact vertebral alignment and no evidence of spondylolisthesis or residual instability (B).

Using Buck's technique, entry points were created at the inferior edge of the lamina, 10 mm lateral to the base of the spinous process. A 3.2 mm drill bit was used to create the screw trajectory, angled 30° laterally to the sagittal plane, passing through the lower pars and into the pedicle. Two 4.5 mm cortical screws were inserted bilaterally across the defects, and autologous bone grafts were packed into the defects to promote fusion and stability. After ensuring hemostasis, the wound was irrigated and closed in layers (Fig. 2A).

Postoperative recovery was uneventful. At one-year follow-up, CT scans revealed resolution of pars defects and maintained vertebral alignment without residual compression or instability (Fig. 2B). There were no surgical complications, and the patient exhibited no neurological deficits. The patient reported significant relief from radicular symptoms and marked improvement in low back pain, allowing for a gradual return to daily activities.

## 3 Discussion

Although the prevalence of spondylolysis with adjacent disc herniation is rare, studies have shown that 3.1 % of patients with L5 spondylolysis had concomitant disc herniation at either L4/L5 or L5/S1 [6]. Spondylolysis creates biomechanical instability, increasing stress on adjacent intervertebral discs; stresses at L4/L5 increase to 111 % in the annulus fibrosus and 120 % in the nucleus pulposus after L5 spondylolysis, while the L5/S1 level experiences even greater axial stresses—168 % and 155 %, respectively [7]. Despite this, degeneration at L4/L5 progresses faster due to hypermobility. In contrast, degeneration at L5/S1 progresses more slowly unless specific sacropelvic parameters predispose it to instability [6,[8], [9], [10]]. This prompt degeneration at L4/L5 is relatively higher than anticipated compared to L5/S1 involvement, though it remains less frequent. This relatively increased prevalence at L4/L5, combined with the preserved range of motion due to hypermobility, makes L4/L5 disc herniation in the setting of L5 spondylolysis particularly challenging to manage.

The management of L5 spondylolysis accompanied by L4/L5 disc herniation requires a comprehensive strategy tailored to the patient's clinical presentation, symptom severity, and response to conservative measures. Conservative treatment remains the first-line approach, particularly for patients with mild to moderate symptoms. This includes physical therapy to strengthen core and lumbar muscles, improve flexibility, and alleviate nerve compression, combined with pain management using Nonsteroidal Anti-Inflammatory Drugs (NSAIDs) and muscle relaxants [11]. Additionally, activity modification is essential to prevent symptom exacerbation, with patients advised to avoid heavy lifting and high-impact activities [12]. In cases of significant instability, bracing with thoracolumbosacral orthosis (TLSO) may help stabilize the spine and limit excessive motion at L4/L5 and L5/S1, although it does not ensure bone union [13]. For persistent or severe symptoms despite conservative management, interventional measures such as epidural steroid injections can provide temporary relief by reducing nerve root inflammation [14].

When conservative treatments fail, surgical intervention becomes necessary. Microdiscectomy, a minimally invasive procedure, effectively relieves nerve compression caused by disc herniation while preserving spinal motion and allowing for a short recovery time. However, it does not address underlying instability from L5 spondylolysis, which, if left untreated, may result in recurrent disc herniation or worsening ASD [6]. The Scott technique, involving bone grafting and direct stabilization of the pars defect with wires or screws, also preserves motion but is less effective in adults over 25 years due to reduced healing capacity and carries a high risk of non-union (up to 18 %), failing to sufficiently prevent long-term ASD [[15], [16], [17]]. Considering the patient's age and the chronic nature of the pars defect, these methods were not suitable for this case.

While lumbar fusion is a definitive solution for spinal instability, it sacrifices motion at the fused level, which can accelerate ASD due to altered biomechanics [18]. In a young, active patient, preserving spinal motion is essential to maintain long-term functionality and avoid premature ASD. Fusion was further deemed unnecessary in this case, as there was no significant spondylolisthesis requiring such intervention [19]. Similarly, the Pedicle Screw-Rod-Hook technique, while effective in stabilizing the pars defect, significantly reduces mobility at the stabilized level, increasing stress on adjacent segments and predisposing to degeneration [20]. The complexity and invasiveness of this method were unnecessary, given the absence of severe instability and the preserved disc integrity at L5/S1. Fusion combined with discectomy could have addressed both instability and nerve root compression, but it would have sacrificed motion at L4/L5 and increased the risk of ASD, a particularly undesirable outcome for a young, active patient.

Buck's technique is a direct repair method for lumbar spondylolysis, involving cortical screw fixation across the pars interarticularis defect, typically reinforced with autologous bone graft. Given the limited literature on optimal management for such cases, this report highlights the importance of individualized treatment strategies to balance spinal stability, motion preservation, and clinical outcomes. In this case, the patient presented with L5 spondylolysis accompanied by L4/L5 disc herniation, a rare combination that required addressing both structural instability and nerve root compression. After careful evaluation, we opted for a combined approach involving Buck's technique and L4/L5 discectomy. This strategy was chosen to achieve nerve root decompression while simultaneously stabilizing the pars defect at L5, all while preserving spinal motion and avoiding unnecessary fusion. Postoperatively, rehabilitation focusing on gradual return to activities, continued physical therapy, and close follow-up is essential to ensure recovery and prevent recurrence. This report also highlights the need for further research to guide individualized treatment strategies for similar complex presentations. Future research should focus on long-term follow-up to assess the durability of the combined surgical approach and to monitor for potential complications, such as ASD, which may impact clinical outcomes over time.

## 4 Conclusion

The combined use of Buck's technique and discectomy addressed the rare coexistence of L5 spondylolysis and L4/L5 disc herniation. By prioritizing motion preservation, this approach resolved spinal instability and nerve root compression, demonstrating significant pain relief and functional recovery. This case highlights the importance of tailored strategies and suggests future research in comparative studies evaluating Buck's technique and fusion for young, active patient cases. It also acknowledges limitations, particularly the lack of long-term follow-up beyond one year.

## CRediT authorship contribution statement

• Shahin Naghizadeh: Investigation, Data collection, Writing - original draft.
• Maryam Zohrabi-Fard: Investigation, Data analysis, Writing - review & editing.
• Saeed Oraee-Yazdani: Conceptualization, Supervision, Data interpretation, Writing - review & editing, Final approval.

## Consent

Written informed consent was obtained from the patient for publication of this case report and accompanying images. A copy of the written consent is available for review by the Editor-in-Chief of this journal on request.

## Ethical approval

Under a constant approval reference, ethical approval for this study was obtained from the relevant institutional ethics committee on 3 October 2023.

## Guarantor

Saeed Oraee-Yazdani accepts full responsibility for the work and the conduct of the study, had access to the data, and controlled the decision to publish.

## Patient perspective

Living with constant lower back pain radiating down my leg transformed even the simplest tasks into daily struggles, and despite months of physiotherapy and trying an array of medications—from anti-inflammatories to strong painkillers—the relief never lasted more than a short while. As my condition worsened, surgery emerged as the only option, and although I was initially anxious, the surgical team's thorough explanations and compassionate communication reassured me that their tailored approach was right for my situation. After the procedure, the early stages of recovery were tough, marked by pain and stiffness, yet I felt an immediate improvement that gave me hope. With their continued support and guidance in the weeks that followed, I steadily regained mobility and watched my quality of life improve. Looking back on the journey, I feel profound gratitude for their expertise, dedication, and the renewed sense of freedom they helped me reclaim.

## Research registration number

1. Name of the registry: Not applicable.
2. Unique identifying number or registration ID: Not applicable.
3. Hyperlink to your specific registration: Not applicable.

## Declaration of Generative AI and AI-assisted technologies in the writing process

During the preparation of this work, the authors used chat GPT in order to paraphrase. After using this tool, the authors reviewed and edited the content as needed and took full responsibility for the content of the publication.

## Funding

This research did not receive any specific grant from funding agencies.

## Declaration of competing interest

None.
